# Perceiving Unpredictability for New Energy Power and Electricity Consumption Forecasting

**DOI:** 10.3390/e28010064

**Published:** 2026-01-05

**Authors:** Lin Zhao, Jian Dong, Ruojing Chen, Yifeng Wang, Yichen Jin, Yi Zhao

**Affiliations:** 1 Institute of Economics and Technology of State Grid Liaoning Electric Power Co., Ltd., Shenyang 110006, China; 2 School of Science, Harbin Institute of Technology, Shenzhen 518055, China

**Keywords:** sensor signal forecasting, unpredictability perception, spectral entropy, adaptive supervision

## Abstract

Accurate prediction of sensor network data in critical domains such as electric power systems and traffic planning is a core task for ensuring grid stability and enhancing urban operational efficiency. Although deep learning models have achieved significant architectural advancements, their training strategy implicitly assumes that all future events are equally predictable, ignoring that the future evolution of sensor signals intertwines deterministic patterns with stochastic events and that prediction difficulty increases with temporal distance. Forcing a model to fit inherently unpredictable events with a uniform supervision may impair its ability to learn generalizable patterns. To address this, we introduce an Unpredictability Perception loss that dynamically computes a supervision weight. The computation of this weight unifies two assessment dimensions of the intrinsic unpredictability of the forecasting task. The first originates from a posterior analysis of the signal content’s randomness, while the second stems from an a priori consideration of temporal distance. The first dimension, through a complexity-aware weight derived from local spectral entropy, reduces supervision on random segments of the signal. The second dimension, through a temporal decay weight based on exponential decay, lessens supervision for distant future points. Applied to the advanced TimeMixer model, experimental results show that our approach achieves performance improvements across multiple public benchmark datasets. By matching the supervision strength to the intrinsic predictability of the signals, our proposed Unpredictability Perception loss function enhances the forecasting accuracy for sensor network data, providing a more reliable technical foundation for ensuring the stability of critical infrastructures like power grids and optimizing urban traffic systems.

## 1. Introduction

Time series forecasting is an indispensable technology in modern scientific and industrial decision-making [1,2,3]. In the domain of energy management, the precise prediction of electricity load, informed by feedback from thousands of sensor nodes within smart grids, serves as the cornerstone for ensuring grid stability, optimizing energy dispatch, and integrating volatile renewable sources such as wind and solar power [4,5,6]. In urban traffic systems, the accurate forecasting of traffic flow, derived from data collected by road sensor networks, provides critical support for intelligent traffic guidance and public resource planning, thereby effectively mitigating daily commuter congestion and responding to traffic disruptions caused by unforeseen incidents [7,8,9]. Similarly, in meteorological science, the long-term prediction of key indicators such as temperature and humidity, monitored by distributed weather sensors, is directly related to agricultural production, extreme weather event warnings, and societal well-being [10,11]. A commonality among these high-stakes applications is the urgent need for models capable of understanding and predicting the high-dimensional time series data generated by massive sensor arrays [12,13]. However, these data themselves are often interwoven with complex dynamic characteristics [14,15], which encompass both learnable, deterministic patterns governed by physical laws or societal rhythms, and are inevitably accompanied by inherently unpredictable, random events triggered by internal system perturbations or abrupt environmental changes [16]. In recent years, the rapid development of deep learning has provided powerful tools to address this challenge. From Transformer-based architectures such as PatchTST [17] and iTransformer [18], to MLP-based models renowned for their simplicity and efficiency like TimeMixer [19], the academic community has made significant progress in enhancing predictive accuracy. These state-of-the-art methods, through their sophisticated structural designs, have far surpassed traditional statistical models in capturing long-term dependencies and complex patterns.

Despite the notable achievements in architectural design, nearly all existing models adhere to a common supervision principle during training, which involves optimizing model parameters by minimizing the Mean Squared Error (MSE) [20]. This supervision principle contains a fundamental assumption that all future events are accurately predictable based on historical information. However, the evolutionary processes of the real world are naturally intertwined with both deterministic patterns and stochastic events. For instance, the electricity load data involved in our experiments contains learnable daily and weekly periodic patterns determined by social production and life rhythms, but it also inevitably includes random disturbances that are inherently unpredictable from historical data, caused by factors such as the random behavior of a large number of individual users, sudden equipment failures, or extreme weather. Likewise, traffic flow data also mixes predictable peak-hour patterns with unpredictable sharp fluctuations caused by contingent events like traffic accidents. The traditional MSE loss function, by imposing a uniform penalty on prediction errors at all future time points, effectively forces the model to fit these intrinsically random and unpredictable future events. This form of supervision may not only lead to the model overfitting to artifacts in the training data but could also impair its ability to learn generalizable underlying patterns, as it compels the model to expend valuable modeling capacity in pursuit of a theoretically unattainable goal.

Therefore, a more effective training paradigm should shift from uniform supervision to adaptive supervision, aligning the learning objective with the intrinsic predictability of future events. This is the core motivation of our research. We posit that the model training process should be redefined as a selective knowledge extraction process rather than a uniform data-fitting task. Specifically, the supervision strength applied to the model should dynamically match the predictability of the task itself. The model should be incentivized to accurately capture predictable dynamics that are close in time and exhibit high signal regularity, while for dynamics that are distant in time or have high signal randomness and thus low predictability, the supervision strength should be appropriately attenuated. Such an adaptive supervision strategy holds the promise of fundamentally optimizing the model’s learning process without adding any architectural complexity, thereby significantly enhancing its robustness and final prediction accuracy.

To this end, we propose an Unpredictability Perception (UP) loss function. This method dynamically computes a supervision weight for each time point in the prediction horizon by fusing a posterior analysis of the signal’s local complexity with a priori knowledge of temporal distance. This weight accurately reflects the intrinsic credibility of each prediction sub-task, thereby guiding the model toward more effective and robust learning. By encoding this inductive bias directly into the optimization objective, we provide a simple yet powerful functional extension to state-of-the-art time series forecasting models, enabling them to make more reliable and precise judgments in complex predictive environments.

## 2. Related Work

### 2.1. Time Series Forecasting Architectures

The field of time series forecasting has undergone a profound evolution from classic statistical methods such as ARIMA and Exponential Smoothing to sophisticated deep learning architectures [21,22,23]. The powerful capabilities of deep learning in capturing complex non-linear dependencies have made it the mainstream approach in recent years. This progression has been primarily driven by innovations in model architecture. Initially, Recurrent Neural Networks (RNNs) and their variants like LSTMs became the foundational models for processing sequential data [24,25,26]. Subsequently, the introduction of Convolutional Neural Networks (CNNs) in models like Temporal Convolutional Network (TCN) demonstrated the effectiveness of causal convolutions in capturing temporal features [27,28].

In recent years, the Transformer architecture, with its self-attention mechanism, has brought about a paradigm shift in the field. The vanilla Transformer [29], however, suffers from quadratic complexity with respect to sequence length, making it inefficient for long-term forecasting. To address this, a series of innovative Transformer variants emerged. Informer introduced the ProbSparse self-attention mechanism and a generative decoder to reduce complexity [30]. Autoformer proposed a decomposition architecture and replaced self-attention with an auto-correlation mechanism [31]. FEDformer further advanced this by leveraging Fourier and Wavelet transforms to perform attention operations in the frequency domain [32]. More recently, PatchTST approached the problem from a new perspective by treating the time series as a sequence of patches [17], similar to Vision Transformers [33], which proved highly effective in capturing local semantic information. Crossformer designed a two-stage attention mechanism to explicitly model cross-time and cross-variable dependencies [34].

While Transformer-based models were rapidly advancing, another line of research began to re-examine the necessity of complex architectures. This led to the emergence of surprisingly simple yet powerful models. DLinear, a straightforward linear model, demonstrated that through proper decomposition, it could outperform many complex Transformer models on several benchmarks [35]. This spurred the development of MLP-based architectures. TimeMixer, a pure multi-layer perceptron model, processes time series across multiple scales and has achieved state-of-the-art performance with remarkable efficiency [19]. Other notable approaches include TimesNet [36], which transforms a 1D time series into a 2D tensor to capture multi-periodicity using a CNN-based method, and MICN [37], which employs multi-scale inception blocks with causal convolutions.

A common thread connects all these architectural explorations. The predominant research focus has been on learning predictable patterns from the past, while overlooking the inherent unpredictability of future events themselves. This standard loss function implicitly assumes that all future events are equally predictable, a premise that does not hold for real-world time series interwoven with deterministic patterns and stochastic events. This highlights a significant research gap, suggesting that optimizing the training objective itself, rather than solely refining the model architecture, offers a promising and underexplored avenue for enhancing forecasting performance.

### 2.2. Adaptive Supervision and Uncertainty Modeling

A parallel stream of research, distinct from architectural design, focuses on the optimization objective itself, particularly on adapting supervision based on data uncertainty. One prominent approach is heteroscedastic regression, which aims to model aleatoric (data) uncertainty [38,39]. These methods typically modify the model’s architecture, for example, by adding a second output head to predict a per-point variance ($\sigma^{2}$). The loss function, often a Gaussian Negative Log-Likelihood ($L\approx \log\left(\sigma^{2}\right)+{\left(y-\hat{y}\right)}^{2}/\sigma^{2}$), then uses this learned, model-dependent variance to down-weight uncertain time points. Another category, often termed confidence-based weighting, focuses on epistemic (model) uncertainty, employing techniques like Monte Carlo Dropout to approximate a Bayesian posterior [40]. The variance of multiple stochastic forward passes is used as an indicator of the model’s confidence.While powerful, these learning-based approaches introduce new challenges. Their efficacy is entirely contingent on the model’s capability to successfully learn the uncertainty itself, which can be an unstable optimization target. Furthermore, they often require modifications to the model architecture (e.g., variance heads) or training process (e.g., stochastic sampling). This suggests a need for an alternative paradigm: an adaptive supervision mechanism that does not depend on a learned uncertainty, but rather on analytically-derived, model-independent properties of the forecasting task.

### 2.3. Causality-Based Forecasting

A distinct and emerging research direction is causality-based forecasting, which moves beyond mere correlation by attempting to model the underlying causal structures that govern time series dynamics [41]. Models in this area aim to identify the true drivers of future events, thereby enhancing robustness against distribution shifts and enabling counterfactual analysis. Recent works have applied these principles to complex, policy-driven domains. For example, Han et al. [42] proposed a causal neural network to improve probabilistic forecasting for carbon prices, leveraging causal insights to handle influencing factors.It is essential to differentiate our UP framework from this causal paradigm. Causality-based models seek to answer why a phenomenon occurs by identifying its structural drivers. Our approach, in contrast, is focused on whether a future event is predictable, based on its own intrinsic signal properties (i.e., local randomness via spectral entropy) and its temporal distance. Therefore, our method is not a causal inference model but rather an associative forecasting model equipped with an analytical, predictability-aware optimization objective. It provides an orthogonal contribution by focusing on the quality of supervision rather than the structural identification of the data generating process.

## 3. Methodology

### 3.1. Framework Overview

**Figure 1:**
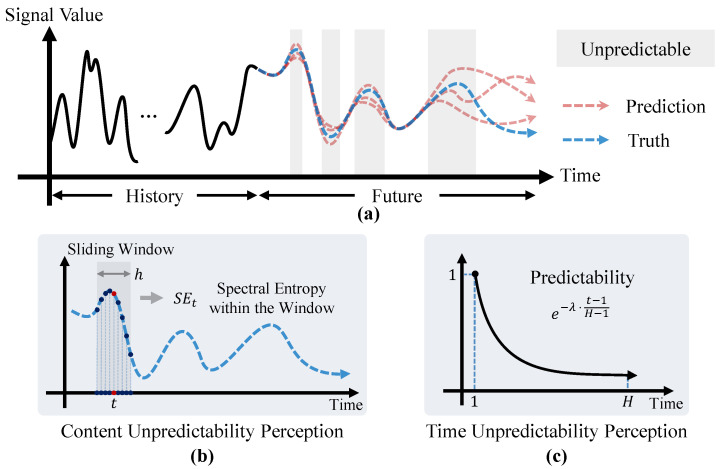
Overview of the Unpredictability Perception loss function. The (**a**) illustrates how future sensor signals contain regions of high unpredictability. The (**b,c**) show the two core components of our method: Content Unpredictability Perception and Time Unpredictability Perception.

Deep learning models have achieved significant success in time series forecasting, largely guided by the optimization of a loss function. The Mean Squared Error (MSE) is widely adopted for its simplicity and ease of optimization. However, a fundamental deficiency of the MSE loss is that it applies a uniform penalty weight to all points within the prediction horizon, failing to differentiate among the intrinsic difficulties of various forecasting tasks. In fact, prediction uncertainty stems from two distinct sources. The first is the inherent randomness of the signal itself, meaning that the sequence dynamics in some time segments are intrinsically harder to predict than in others. The second is the a priori difficulty of the forecasting task, where predictions for the distant future naturally contain more uncertainty than those for the near future. An advanced training paradigm should be able to recognize and adapt to both types of uncertainty, thereby intelligently allocating the model’s learning resources to patterns that are genuinely learnable in both a content and temporal sense. To this end, we propose the UP loss function, which guides the model toward more effective and robust learning by dynamically computing a supervision weight for each time point in the prediction horizon. The construction of this weight integrates a posterior analysis of the signal’s local complexity with a priori knowledge of temporal distance, as conceptually illustrated in Figure 1.

**Figure 2:**
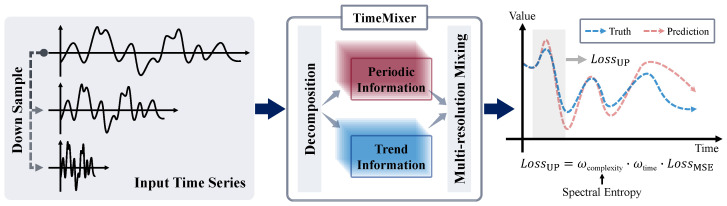
Systematic framework illustrating the integration of the UP Loss with a backbone forecasting model. The input time series is processed by the backbone model (e.g., TimeMixer using decomposition and mixing) to generate the prediction. The UP Loss module then compares the prediction to the ground truth. It computes the final $L_{\mathrm{UP}}$ by weighting the standard MSE, using $w_{\mathrm{complexity}}$ (derived from spectral entropy) and $w_{\mathrm{time}}$ (derived from temporal distance), to guide backpropagation.

To elucidate the complete computational flow and the integration of our loss function with a backbone forecasting model, we present a systematic framework diagram in Figure 2. As depicted, the input time series is first processed by the core forecasting model (e.g., TimeMixer), which utilizes its internal architecture, such as decomposition and multi-resolution mixing, to generate the prediction sequence $\hat{Y}$. The proposed UP Loss module then operates as the optimization objective. It takes both the model’s prediction $\hat{Y}$ and the ground-truth sequence *Y* as inputs. Critically, it computes the complexity weight ($w_{\mathrm{complexity}}$) by analyzing the spectral entropy of the ground-truth signal itself, while concurrently calculating the temporal decay weight ($w_{\mathrm{time}}$). These two weights are multiplicatively combined to modulate the standard Mean Squared Error, forming the final $L_{\mathrm{UP}}$. This adaptively weighted loss is then used to compute the gradients and update the parameters of the forecasting model via backpropagation.

### 3.2. Local Complexity Quantification via Spectral Entropy

The first component of our method aims to quantify the predictability of the signal content itself. This addresses what is formally known as aleatoric uncertainty, the irreducible randomness inherent in the data generation process. In time series analysis, particularly in volatile energy systems, segments dominated by aleatoric uncertainty (e.g., sensor noise or chaotic wind power fluctuations) theoretically lack extractable patterns. Forcing a model to fit these segments leads to overfitting. Therefore, we require a metric to distinguish between deterministic patterns and stochastic noise.

Ideally, **Kolmogorov complexity** from algorithmic information theory offers the ultimate theoretical measure of randomness, defining the complexity of a sequence as the length of its shortest generative program [43,44]. However, since this complexity is theoretically uncomputable, it inspires the search for an effective and computable proxy. We employ **Spectral Entropy (SE)** [45] for this purpose. By calculating the Shannon entropy of a signal’s power spectral density, spectral entropy effectively measures the signal’s structural regularity rather than just its amplitude [46,47]. A signal segment with a simple, regular structure has its energy concentrated in a few frequencies, resulting in low spectral entropy and indicating high predictability. Conversely, a segment resembling white noise has its energy uniformly distributed across the frequency domain, yielding high spectral entropy and indicating weak predictability. This makes SE an ideal, theoretically grounded proxy for modulating supervision based on inherent signal learnability.

To capture these time-varying local dynamics, we adopt a sliding window strategy. For any time point t∈{1,…,H} within the ground-truth future sequence $Y=(y_{1},y_{2},\ldots ,y_{H})$, we define a local window $Y_{t}^{\mathrm{local}}$ of length $P_{\mathrm{local}}$ centered at that point. To compute its spectral entropy, we first estimate the Power Spectral Density (PSD). The signal within the window is processed by the Fast Fourier Transform (FFT) to obtain its frequency representation. The periodogram, a common estimator for the PSD, is then calculated as

(1) $$P_{t}\left(f\right)=\frac{1}{P_{\mathrm{local}}}{\left|\sum_{n=0}^{P_{\mathrm{local}}-1}y_{t,n}^{\mathrm{local}}e^{-j2\pi fn/P_{\mathrm{local}}}\right|}^{2}$$

where $y_{t,n}^{\mathrm{local}}$ is the *n*-th sample in the window $Y_{t}^{\mathrm{local}}$ and *f* represents the discrete frequency bins. To treat the power distribution as a probability distribution, we normalize the PSD across all *K* frequency bins

(2) $$p_{t}\left(f_{k}\right)=\frac{P_{t}\left(f_{k}\right)}{\sum_{i=1}^{K}P_{t}\left(f_{i}\right)}$$

The Shannon entropy of this distribution is then computed as

(3) $$H\left(p_{t}\right)=-\sum_{k=1}^{K}p_{t}\left(f_{k}\right)\log_{2}\left(p_{t}\left(f_{k}\right)\right)$$

To ensure the entropy value is scaled between 0 and 1, facilitating its use as a weight, we normalize it by the maximum possible entropy for a *K*-bin distribution, which is $\log_{2}\left(K\right)$. This yields the final Local Spectral Entropy $SE_{t}$:

(4) $$SE_{t}=\frac{H(p_{t})}{\log_{2}\left(K\right)}$$

From this, we define the first weight component, the complexity weight $w_{\mathrm{complexity},t}$, which is inversely proportional to the signal’s randomness:

(5) $$w_{\mathrm{complexity},t}=1-SE_{t}$$

This weight assigns a value approaching 1 to time points in regular, predictable regions and a value approaching 0 to those in noisy, random regions. This mechanism compels the model during training to focus its attention more on signal segments that exhibit clear and learnable patterns.

### 3.3. Temporal Weighting via Exponential Decay

The second component addresses epistemic uncertainty, which arises from the model’s lack of knowledge about the future state. A fundamental axiom in forecasting is that this uncertainty accumulates as the prediction horizon extends, often referred to as the “butterfly effect” in dynamical systems. To model this horizon-dependent uncertainty, we introduce a temporal decay weight $w_{\mathrm{time},t}$. We specifically select an exponential decay function, $w\left(t\right)=e^{-\lambda t}$, rather than a linear one, to align with the information-theoretic principle that the mutual information between the current state and the future state decays exponentially in chaotic or mixing systems. This non-linear formulation effectively penalizes errors in the near-term (where high precision is feasible) while gracefully relaxing constraints in the long-term (where uncertainty is dominant), thereby aligning the optimization objective with the theoretical limits of predictability.

(6) $$w_{\mathrm{time},t}=\exp\left(-\lambda \cdot \frac{t-1}{H-1}\right)$$

where *t* is the time step index in the prediction horizon, ranging from 1 to *H*. The term $\frac{t-1}{H-1}$ normalizes the time step to the interval from 0 to 1, ensuring that the decay process is independent of the specific forecast horizon and thus preserving the generalizability of the mechanism across different tasks. λ is a non-negative hyperparameter that controls the rate of decay. When λ is zero, temporal decay has no effect, and the model treats all time points equally regardless of their distance. When λ is greater than zero, more distant prediction points are assigned a stronger decay in their weights.

The final supervision weight $w_{\mathrm{final},t}$ is determined by the element-wise product of the complexity and temporal decay weights. This multiplicative fusion integrates two distinct dimensions of unpredictability assessment, one derived from a posteriori analysis of the signal and the other from a priori temporal knowledge, ensuring that a high supervision strength is assigned only to time points that are concurrently characterized by high signal regularity and temporal proximity.

(7) $$w_{\mathrm{final},t}=w_{\mathrm{complexity},t}\cdot w_{\mathrm{time},t}=\left(1-SE_{t}\right)\cdot \exp\left(-\lambda \cdot \frac{t-1}{H-1}\right)$$

Based on this dynamic weight, we construct the final UP loss function. Given the model’s predicted sequence $\hat{Y}=\left({\hat{y}}_{1},{\hat{y}}_{2},\ldots ,{\hat{y}}_{H}\right)$, the UP loss is calculated as

(8) $$L_{\mathrm{UP}}=\frac{1}{H}\sum_{t=1}^{H}w_{\mathrm{final},t}\cdot {\left(y_{t}-{\hat{y}}_{t}\right)}^{2}$$

This loss function, while operationally simple, profoundly alters the model’s learning paradigm. It guides the model to preferentially allocate its finite modeling capacity to recent and regular portions of the signal, while permitting greater error on distant or inherently noisy segments. This dual-dimension adaptive regularization aims to produce a model that is not only more accurate but also more aligned with the fundamental principles of inductive inference in its very design.

### 3.4. Algorithmic Implementation and Computational Flow

To ensure methodological clarity and reproducibility, we formalize the complete training procedure, which integrates the Unpredictability Perception loss with a backbone forecasting model *M*. To clarify the integration of our method, let D={(X,Y)} denote the dataset, where *X* is the lookback window and *Y* is the forecast horizon. Let $f_{\theta }\left(\cdot \right)$ represent the backbone forecasting model (e.g., TimeMixer) parameterized by θ. Our UP loss replaces the standard loss function during the backward pass. The complete training procedure, detailing the interaction between the backbone model’s forward pass and the UP loss’s weight calculation, is formalized in Algorithm 1.
**Algorithm1:** Training Procedure with Unpredictability Perception (UP) Loss**Require:** Training dataset $D={\left\{\left(X_{i},Y_{i}\right)\right\}}_{i=1}^{N}$, Forecasting Model $f_{\theta }$ with parameters θ **Require:** Hyperparameters: Learning rate η, Local window size $P_{\mathrm{local}}$, Temporal decay factor λ, Max epochs *E* **Ensure:**  Optimized model parameters $\theta^{*}$
  1:  Initialize θ randomly  2:  **for** epoch e=1 to *E* **do**  3:      **for** each batch (X,Y) sampled from D **do**  4:          $\hat{Y}\leftarrow f_{\theta }\left(X\right)$ Forward pass  5:          H←Length(Y)  6:          Initialize weight vector $W_{\mathrm{final}}\in R^{H}$  7:          **for** t=1 to *H* **do**  8:              $Y_{t}^{\mathrm{local}}\leftarrow \mathrm{ExtractLocalWindow}\left(Y,t,P_{\mathrm{local}}\right)$  9:              $SE_{t}\leftarrow \mathrm{ComputeSpectralEntropy}\left(Y_{t}^{\mathrm{local}}\right)$10:              $w_{\mathrm{complexity},t}\leftarrow 1-SE_{t}$11:              $w_{\mathrm{time},t}\leftarrow \exp\left(-\lambda \cdot \frac{t-1}{H-1}\right)$12:              $w_{\mathrm{final}}\left[t\right]\leftarrow w_{\mathrm{complexity},t}\cdot w_{\mathrm{time},t}$13:          **end for**14:          $L_{\mathrm{UP}}\leftarrow \frac{1}{H}\sum_{t=1}^{H}w_{\mathrm{final}}\left[t\right]\cdot {\left(Y\left[t\right]-\hat{Y}\left[t\right]\right)}^{2}$15:          $\theta \leftarrow \theta -\eta \cdot \nabla_{\theta }L_{\mathrm{UP}}$ Update model parameters (Backpropagation)16:      **end for**17:**end for**18:**return **$\theta^{*}$


The framework is designed to be model-agnostic, allowing *M* to be any forecasting architecture.The algorithm commences by obtaining a prediction $\hat{Y}$ from the model’s forward pass. It then iterates through the prediction horizon *H* to compute the two core weight components for each time step *t*. The complexity weight $w_{\mathrm{complexity},t}$ is derived by analyzing the local spectral entropy $SE_{t}$ of the ground-truth signal *Y*, as detailed in Section 2.2. Concurrently, the temporal weight $w_{\mathrm{time},t}$ is calculated based on the exponential decay function described in Section 2.3. These two weights are multiplicatively fused to create the final weight $w_{\mathrm{final},t}$. This final weight dynamically scales the point-wise squared error. The mean of these weighted errors constitutes the final $L_{\mathrm{UP}}$, which is then used for backpropagation to optimize the model’s parameters. Key hyperparameters in this process include the local window size $P_{\mathrm{local}}$ for spectral entropy calculation and the decay rate λ for temporal weighting. We set $P_{\mathrm{local}}=24$ and λ=0.5 as a robust default.

### 3.5. Computational Complexity Analysis

A critical consideration for any novel loss function is the potential introduction of computational overhead. The standard MSE loss has a time complexity of O(H) per sample, where *H* is the prediction horizon. Our proposed UP loss introduces two additional weight calculation steps. The temporal decay weight involves a constant-time operation O(1) per time step. The complexity weight requires computing the Spectral Entropy, which relies on the Fast Fourier Transform (FFT) applied to a local window of size $P_{\mathrm{local}}$. The complexity of the FFT operation is $O(P_{\mathrm{local}}\log P_{\mathrm{local}})$. Consequently, the total complexity of the UP loss calculation for one time step is $O(P_{\mathrm{local}}\log P_{\mathrm{local}})$.

It is important to note that since the calculation of weights depends solely on the ground truth *Y*, which remains static during training, these weights can theoretically be pre-computed offline, reducing the online training overhead to zero. However, to evaluate the worst-case scenario, we assessed the runtime cost when computing weights on-the-fly during training. We conducted a comparative experiment between the original TimeMixer and our proposed UP-TimeMixer under identical hardware and software configurations (NVIDIA RTX 4090 GPU, Batch Size 32, Input/Output Horizon 96). As detailed in Table 1, the theoretical time complexity of the UP loss calculation for one time step is $O(P_{\mathrm{local}}\log P_{\mathrm{local}})$. In practice, this introduces a marginal overhead during the training phase. The average training time per epoch increased from 11.23±0.15 s (TimeMixer) to 11.68±0.18 s (UP-TimeMixer), representing a relative increase of approximately 4.0%. This slight increase is attributable to the on-the-fly FFT computation for spectral entropy. Crucially, however, the proposed UP loss functions exclusively as an optimization objective and is detached from the model architecture after training. Consequently, the inference time remains strictly identical (1.42±0.03 ms per batch), and the model parameter count is unchanged. This confirms that our method improves predictive accuracy without imposing any additional computational burden during the deployment phase, making it highly suitable for latency-sensitive applications.

## 4. Experiments and Results

### 4.1. Datasets

To comprehensively evaluate the effectiveness and generalization capability of our proposed framework, we selected eight public benchmark datasets covering critical domains such as energy, meteorology, and traffic. These datasets exhibit significant diversity in their temporal characteristics, providing an ideal testbed for validating the core ideas of our method.

The ETT series (ETTh1, ETTh2, ETTm1, and ETTm2) [48] records key metrics of power transformers. This data is known for its clear seasonal periodicity but is also interspersed with substantial random noise introduced by grid load fluctuations and environmental factors. This coexistence of periodicity and randomness constitutes a perfect scenario for testing a model’s ability to distinguish between predictable patterns and unpredictable disturbances.

The Weather dataset contains 21 meteorological indicators. A meteorological system is inherently a complex chaotic system, with its long-term dynamics being theoretically unpredictable. In the short term, however, it exhibits discernible patterns governed by geophysical laws. This dataset offers an opportunity to test our model’s performance in a complex dynamical system with intrinsic prediction limits.

The Electricity dataset records the hourly electricity consumption of 321 clients. Its dynamics are dominated by strong periodic patterns determined by social production and life, such as daily and weekly rhythms. However, the aggregation of random behaviors from a massive number of individual users injects continuous random disturbances into this macroscopic regularity, which are difficult to model from historical data alone.

The Traffic dataset describes the occupancy rates of road sensors in the San Francisco Bay Area. Traffic flow data is a typical dual-characteristic signal; it contains predictable peak and off-peak patterns driven by commuting regularities, yet it is frequently impacted by sudden, contingent events like traffic accidents, which produce unforeseen sharp fluctuations.

The Solar-Energy dataset [49] records the power generation of 137 solar power plants. Photovoltaic power generation is directly subject to highly variable meteorological factors such as cloud cover and light intensity, causing the signal to exhibit extreme volatility and intermittency. The signal contains a large number of inherently unpredictable segments driven by the randomness of weather systems.

These datasets, sourced from various sensor networks, all exhibit an interweaving of deterministic patterns and stochastic fluctuations, providing a rigorous empirical foundation for evaluating the effectiveness of the proposed framework.

### 4.2. Experimental Setup

To verify the practical value of our proposed framework, we chose to integrate and validate it on TimeMixer, one of the current state-of-the-art models. As a pure MLP-based architecture, TimeMixer has achieved SOTA performance on numerous time series forecasting benchmarks, surpassing many structurally complex Transformer models. The pure MLP architecture of TimeMixer lacks sequence-specific inductive biases, making it particularly sensitive to the quality of the supervision signal. Our proposed optimization framework provides the precise guidance it needs to distinguish between deterministic patterns and random noise.

In all experiments, we evaluated all models on four progressively longer prediction tasks of 96, 192, 336, and 720 steps. To ensure a fair comparison and reproducibility, our experimental setup strictly aligns with the configurations of the baseline TimeMixer model. Specifically, all models were implemented using PyTorch 2.4.1 and trained on a server equipped with two NVIDIA RTX 4090 GPUs. Following the official implementation of TimeMixer, we utilized the Adam optimizer with a learning rate adjusted between $10^{-3}$ and $10^{-2}$ depending on the dataset characteristics, and a batch size ranging from 32 to 128. An early stopping mechanism with a patience of 10 epochs was employed to prevent overfitting. The local window size $P_{\mathrm{local}}$ was set to 24 for hourly datasets. The calculation of Spectral Entropy relies on the frequency domain representation derived via the Discrete Fourier Transform (DFT). A window size of 24 provides the minimal sufficient spectral resolution (approximately 12 effective frequency bins up to the Nyquist limit) to generate a statistically stable entropy estimate. Smaller windows (e.g., 12 points) result in a sparse spectrum, leading to mathematically unstable entropy values that fail to distinguish noise from signal. Conversely, significantly larger windows risk smoothing out the local volatility that our method aims to capture. Furthermore, this setting maintains consistency with the moving average kernel size used in the backbone TimeMixer model, ensuring structural alignment. For the temporal decay factor, we set λ=0.5 as a fixed default across all experiments. This parameter alignment strategy ensures that the performance improvements originate from the proposed method’s mechanism rather than extensive hyperparameter tuning. The source code will be made publicly available upon acceptance to promote uncertainty awareness in deep learning models.

### 4.3. Quantitative Comparison

Detailed experimental results are presented in Table 2 and Table 3. To thoroughly assess the performance of our method, we divided the comparison models into two groups based on their publication dates: the first group includes recent state-of-the-art models that are contemporary competitors, while the second group consists of influential and representative models from the past several years. The experimental data shows that our method achieves stable and significant performance improvements over the original TimeMixer model and sets new state-of-the-art performance records in the vast majority of test scenarios.

The performance gains achieved by our method are particularly prominent on the ETT series and Solar-Energy datasets, which are characterized by the coexistence of periodicity and noise. The signals in these datasets contain a large amount of endogenous randomness. The traditional MSE loss forces the model to fit this unlearnable noise, thereby impairing its ability to capture core periodic patterns. Our UP loss function, through its complexity-aware mechanism, effectively reduces the supervision weight on these highly random signal segments. This allows the model to focus more of its modeling capacity on learning the underlying patterns that have genuine generalization value, leading to a substantial improvement in prediction accuracy.

On the Weather, Electricity, and Traffic datasets, the results exhibit different characteristics. The signal dynamics in these datasets are dominated by energy-concentrated, low-frequency periodic components. This causes the local spectral entropy of the signal to remain at a low level for most of the time, thereby weakening the differential adjustment capability of the complexity weight $w_{complexity,t}$ in UP-TimeMixer and making the supervision signal it provides somewhat approximate that of standard MSE. However, the prior knowledge that prediction difficulty increases with the time step still holds true in these tasks. Therefore, the temporal decay weight $w_{time,t}$ in UP-TimeMixer plays a key regularization role. Especially in long-term forecasting tasks, by reducing the supervision strength on distant future points, it effectively prevents the model from overfitting in regions of higher uncertainty. This explains why, although the advantage of our method is less pronounced on these datasets, it still consistently outperforms others in most long-term forecasting scenarios. It also demonstrates the complementarity and robustness of the two weight components designed within the UP-TimeMixer.

Ultimately, the experimental results indicate that guiding a model’s learning process through a more refined optimization objective is an equally effective, and perhaps even more efficient, path to improving prediction performance than continually designing more complex model architectures. A simple MLP model, when equipped with our unpredictability perception framework, can systematically outperform numerous structurally complex Transformer models. This offers an inspiring new perspective for the future development of the time series forecasting field.

### 4.4. Visualization Comparison

**Figure 3:**
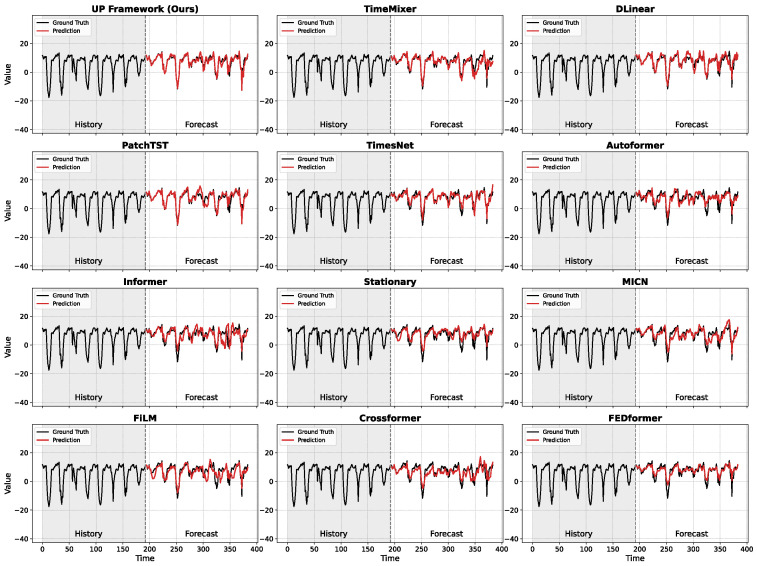
Visual comparison of different time series prediction models on a signal segment from the ETTh1 dataset. The grey shaded region denotes the lookback window (historical input), while the dashed vertical line marks the onset of the forecasting horizon. Ground truth is shown in black, with model predictions displayed in color.

A qualitative analysis of the forecast results, as visually presented in Figure 3, reveals that the uniform training objectives of conventional models result in characteristic failure modes. For instance, earlier Transformer models like Informer and Autoformer tend to produce erratic, high-amplitude oscillations decorrelated from the ground truth. This suggests that forcing a model to equally fit all future points, including inherently unpredictable ones, can impair the learning of stable long-term patterns. Models with strong smoothing biases, such as DLinear and TimesNet, exhibit another reaction to this uniform objective by systematically dampening the amplitude of sharp peaks, effectively prioritizing more predictable low-frequency trends over volatile, high-frequency details. Even robust architectures like PatchTST may occasionally produce spurious, high-amplitude patterns, a behavior suggesting that a uniform training objective may cause overfitting to locally complex yet globally insignificant features. The baseline TimeMixer successfully captures the signal’s primary dynamics and periodicities, but its prediction curve contains excessive high-frequency oscillations, indicating its uniform training objective may have misinterpreted random noise as an intrinsic signal pattern to be fitted. Our proposed UP-TimeMixer, however, visibly overcomes this specific limitation, yielding a prediction that is both structurally accurate and significantly less noisy. By encoding signal predictability and temporal distance into its loss function, our method mitigates the inherent limitations of uniform supervision. The model’s capacity to precisely approximate both low-frequency trends and high-frequency transients demonstrates the efficacy of this adaptive training paradigm. By concentrating modeling resources on learnable signal components, it effectively prevents overfitting to stochastic, unpredictable dynamics.

### 4.5. Ablation Study

To evaluate the contribution of each component of the proposed UP Loss, we conduct an ablation study across all eight datasets used in our experiments. We compare the baseline model (using only the standard MSE loss) with three variants: one using only the temporal weight $w_{\mathrm{time}}$, one using only the complexity weight $w_{\mathrm{complexity}}$, and the full UP Loss that combines both components. The results, summarized in Table 4, show that the full UP-TimeMixer consistently outperforms the baseline MSE in most cases, confirming the effectiveness of re-weighting the loss function. The most significant improvements are observed in datasets such as ETTh1, ETTm2, and Solar-Energy, where the full UP Loss reduces the average MSE and MAE by a small but consistent margin.

However, the impact of the individual components of the UP Loss varies across datasets. On datasets like ETTh2 and ETTm1, where horizon-dependent uncertainty is more pronounced, the temporal weight $w_{\mathrm{time}}$ contributes more to the reduction in error than the complexity weight $w_{\mathrm{complexity}}$. For instance, on ETTh2 at the 720-step horizon, using only $w_{\mathrm{time}}$ yields a better MSE than using only $w_{\mathrm{complexity}}$. On the other hand, for datasets with more regular periodicity, such as Solar-Energy and ETTh1, the complexity weight $w_{\mathrm{complexity}}$ proves to be more beneficial. For example, on ETTh1 at the 96-step horizon, the complexity-based model performs slightly better than the temporal-only variant. Interestingly, the full UP Loss does not always outperform the baseline on datasets with some predictable signals, such as Weather and Electricity. In these cases, the addition of re-weighting does not result in substantial gains, and the baseline MSE performs comparably or even slightly better.

**Figure 4:**
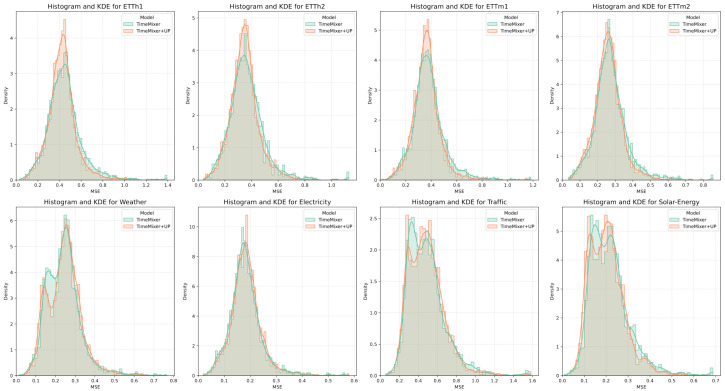
Histograms and kernel density estimates of the mean squared error distributions for the baseline TimeMixer model (teal) and TimeMixer with Unpredictability Perception loss (orange) across all benchmark datasets.

To visualize the contribution of our proposed Unpredictability Perception loss, we compare the empirical distributions of mean squared errors between the baseline TimeMixer and TimeMixer+UP on the test sets. Figure 4 presents histograms overlaid with kernel density estimates (KDE) for the baseline TimeMixer and TimeMixer+UP, derived from per-instance MSE values aggregated over the prediction horizons (96, 192, 336, 720 steps). In datasets such as ETTh1, ETTh2, ETTm1, ETTm2, and Solar-Energy, where TimeMixer+UP yields lower average MSE, the distributions exhibit modes centered at reduced error levels, often accompanied by narrower spreads and diminished high-error tails, reflecting improved generalization and robustness to stochastic signal components. Overall, these visualizations affirm the efficacy of the UP loss in modulating supervision to match the intrinsic predictability of sensor signals, thereby fostering more stable and accurate forecasts in critical applications like power grid management and urban traffic optimization.

This ablation study demonstrates that the full UP Loss generally improves forecasting performance by effectively re-weighting the loss to prioritize more predictable segments. The individual components (temporal and complexity weights) have different impacts depending on the dataset, with the temporal weight being more effective on datasets with higher uncertainty and the complexity weight being more useful on datasets with regular patterns. These findings validate the proposed loss function and highlight its adaptability across different types of time series data.

To rigorously validate the selection of the hyperparameter λ=0.5, we conducted a comprehensive parameter sensitivity analysis on the representative ETTh1 dataset. We evaluated the model’s performance by varying λ across the set {0.1,0.3,0.5,0.7,0.9} while keeping all other parameters fixed. The results, summarized in Table 5, reveal a distinct pattern. When λ is small, the temporal weight function approximates a uniform distribution, resulting in a supervision signal similar to the standard MSE; consequently, the performance improvement over the baseline is marginal. As λ increases to 0.5, the loss function effectively down-weights the supervision for distant, high-uncertainty time steps, allowing the model to prioritize learnable short-term dynamics, which leads to the lowest average MSE of 0.436. However, as λ continues to increase beyond 0.7, the excessive decay overly relaxes the constraints on long-term predictions. This causes the model to essentially neglect the supervision for the distant future, resulting in a degradation of overall forecasting accuracy. This empirical evidence confirms that λ=0.5 provides the optimal equilibrium between enforcing prediction accuracy and accommodating temporal uncertainty.

## 5. Conclusions

This study introduced an Unpredictability Perception Framework to address the inherent limitations of the mean squared error loss function in time series forecasting. Our approach redefines the model’s optimization objective through an innovative loss function that dynamically computes supervision weights based on a dual assessment of the forecasting task’s intrinsic unpredictability. This assessment unifies a posterior analysis of signal content randomness via local spectral entropy with an a priori consideration of temporal distance via exponential decay. This mechanism guides the model to prioritize learning high-certainty deterministic patterns while reducing the fitting intensity on inherently random or distant uncertain events. By applying this framework to the advanced TimeMixer model, experimental results across multiple public benchmarks demonstrate that this optimization at the loss function level systematically enhances prediction accuracy without any architectural modifications. The findings of this research reveal that matching the supervision strength to the intrinsic predictability of the forecasting task provides a simple yet effective path toward enhancing the robustness and accuracy of deep learning forecasting models, offering a new optimization perspective for sensor network data analysis. 

## Figures and Tables

**Table 1:** Comparison of computational complexity and actual running time on the ETTh1 dataset (Input-96-Predict-96). Inference time is measured per batch (size = 32).

Model	Training Complexity	Training Time (s/Epoch)	Inference Time (ms/Batch)	Params
TimeMixer	O(L)	11.23±0.15	1.42±0.03	24 K
UP-TimeMixer	O(L+H·PlogP)	11.68±0.18	1.42±0.03	24 K

**Table 2:** Performance comparison with contemporary state-of-the-art models for time series forecasting. The best results are in bold, and the second best are underlined.

Models		UP-TimeMixer (Ours)		TimeMixer 2024		PatchTST 2023		TimesNet 2023		Crossformer 2023		DLinear 2023	
Metric		MSE	MAE	MSE	MAE	MSE	MAE	MSE	MAE	MSE	MAE	MSE	MAE
ETTh1	96	**0.371**	**0.395**	0.375	0.400	0.460	0.447	0.384	0.402	0.423	0.448	0.397	0.412
	192	**0.423**	**0.415**	0.429	0.421	0.512	0.477	0.436	0.429	0.471	0.474	0.446	0.441
	336	**0.472**	**0.449**	0.484	0.458	0.546	0.496	0.638	0.469	0.570	0.546	0.489	0.467
	720	**0.479**	**0.465**	0.498	0.482	0.544	0.517	0.521	0.500	0.653	0.621	0.513	0.510
	Avg	**0.436**	**0.431**	0.447	0.440	0.516	0.484	0.495	0.450	0.529	0.522	0.461	0.457
ETTh2	96	**0.285**	**0.338**	0.289	0.341	0.308	0.355	0.340	0.374	0.745	0.584	0.340	0.394
	192	**0.365**	**0.385**	0.372	0.392	0.393	0.405	0.402	0.414	0.877	0.656	0.482	0.479
	336	**0.381**	**0.409**	0.386	0.414	0.427	0.436	0.452	0.452	1.043	0.731	0.591	0.541
	720	**0.405**	**0.428**	0.412	0.434	0.436	0.450	0.462	0.468	1.104	0.763	0.839	0.661
	Avg	**0.359**	**0.390**	0.364	0.395	0.391	0.411	0.414	0.427	0.942	0.684	0.563	0.519
ETTm1	96	**0.315**	**0.351**	0.320	0.357	0.352	0.374	0.338	0.375	0.404	0.426	0.346	0.374
	192	**0.355**	**0.375**	0.361	0.381	0.390	0.393	0.374	0.387	0.450	0.451	0.382	0.391
	336	**0.382**	**0.398**	0.390	0.404	0.421	0.414	0.410	0.411	0.532	0.515	0.415	0.415
	720	**0.441**	**0.432**	0.454	0.441	0.462	0.449	0.478	0.450	0.666	0.589	0.473	0.451
	Avg	**0.373**	**0.389**	0.381	0.395	0.406	0.407	0.400	0.406	0.513	0.495	0.404	0.408
ETTm2	96	**0.171**	**0.255**	0.175	0.258	0.183	0.270	0.187	0.267	0.287	0.366	0.193	0.293
	192	**0.232**	**0.294**	0.237	0.299	0.255	0.314	0.249	0.309	0.414	0.492	0.284	0.361
	336	**0.291**	**0.335**	0.298	0.340	0.309	0.347	0.321	0.351	0.597	0.542	0.382	0.429
	720	**0.382**	**0.390**	0.391	0.396	0.412	0.404	0.408	0.403	1.730	1.042	0.558	0.525
	Avg	**0.269**	**0.319**	0.275	0.323	0.290	0.334	0.291	0.333	0.757	0.610	0.354	0.402
Weather	96	0.179	0.232	**0.163**	**0.209**	0.186	0.227	0.172	0.220	0.195	0.237	0.195	0.252
	192	0.221	0.264	**0.208**	**0.250**	0.234	0.265	0.219	0.261	0.209	0.277	0.237	0.295
	336	0.265	0.304	0.251	**0.287**	0.284	0.301	**0.246**	0.337	0.273	0.332	0.282	0.331
	720	0.349	0.358	**0.339**	**0.341**	0.356	0.349	0.365	0.359	0.379	0.401	0.345	0.382
	Avg	0.254	0.290	**0.240**	**0.271**	0.265	0.285	0.251	0.294	0.264	0.320	0.265	0.315
Electricity	96	0.165	0.257	**0.153**	**0.247**	0.190	0.296	0.168	0.272	0.219	0.314	0.210	0.302
	192	0.173	0.263	**0.166**	**0.256**	0.199	0.304	0.184	0.322	0.231	0.322	0.210	0.305
	336	**0.182**	**0.273**	0.185	0.277	0.217	0.319	0.198	0.300	0.246	0.337	0.223	0.319
	720	**0.219**	**0.308**	0.225	0.310	0.258	0.352	0.220	0.320	0.280	0.363	0.258	0.350
	Avg	0.185	0.275	**0.182**	**0.272**	0.216	0.318	0.193	0.304	0.244	0.334	0.225	0.319
Traffic	96	0.470	0.287	**0.462**	**0.285**	0.526	0.347	0.593	0.321	0.644	0.429	0.650	0.396
	192	0.479	**0.292**	**0.473**	0.296	0.522	0.332	0.617	0.336	0.665	0.431	0.598	0.370
	336	**0.492**	**0.294**	0.498	0.296	0.517	0.334	0.629	0.336	0.674	0.420	0.605	0.373
	720	**0.501**	**0.309**	0.506	0.313	0.552	0.352	0.640	0.350	0.683	0.424	0.645	0.394
	Avg	0.486	**0.296**	**0.484**	0.297	0.529	0.341	0.620	0.336	0.667	0.426	0.625	0.383
Solar-Energy	96	**0.186**	**0.256**	0.189	0.259	0.265	0.323	0.373	0.358	0.232	0.302	0.290	0.378
	192	**0.218**	**0.279**	0.222	0.283	0.288	0.332	0.397	0.376	0.371	0.410	0.320	0.398
	336	**0.227**	**0.288**	0.231	0.292	0.301	0.339	0.420	0.380	0.495	0.515	0.353	0.415
	720	**0.219**	**0.281**	0.223	0.285	0.295	0.336	0.420	0.381	0.526	0.542	0.357	0.413
	Avg	**0.213**	**0.276**	0.216	0.280	0.287	0.333	0.403	0.374	0.406	0.442	0.330	0.401

**Table 3:** Performance comparison with representative models from the last five years for time series forecasting. The best results are in bold, and the second best are underlined.

Models		UP-TimeMixer (Ours)		MICN 2022		FiLM 2022		FEDformer 2022		Stationary 2022		Autoformer 2021		Informer 2021	
Metric		MSE	MAE	MSE	MAE	MSE	MAE	MSE	MAE	MSE	MAE	MSE	MAE	MSE	MAE
ETTh1	96	**0.371**	**0.395**	0.426	0.446	0.438	0.433	0.395	0.424	0.513	0.491	0.449	0.459	0.865	0.713
	192	**0.423**	**0.415**	0.454	0.464	0.493	0.466	0.469	0.470	0.534	0.504	0.500	0.482	1.008	0.792
	336	**0.472**	**0.449**	0.493	0.487	0.547	0.495	0.530	0.499	0.588	0.535	0.521	0.496	1.107	0.809
	720	**0.479**	**0.465**	0.526	0.526	0.586	0.538	0.598	0.544	0.643	0.616	0.514	0.512	1.181	0.865
	Avg	**0.436**	**0.431**	0.475	0.480	0.516	0.483	0.498	0.484	0.570	0.537	0.496	0.487	1.040	0.795
ETTh2	96	**0.285**	**0.338**	0.372	0.424	0.322	0.364	0.358	0.397	0.476	0.458	0.346	0.388	3.755	1.525
	192	**0.365**	**0.385**	0.492	0.492	0.404	0.414	0.429	0.439	0.512	0.493	0.456	0.452	5.602	1.931
	336	**0.381**	**0.409**	0.607	0.555	0.435	0.445	0.496	0.487	0.552	0.551	0.482	0.486	4.721	1.835
	720	**0.405**	**0.428**	0.824	0.655	0.447	0.458	0.463	0.474	0.562	0.560	0.515	0.511	3.647	1.625
	Avg	**0.359**	**0.390**	0.574	0.531	0.402	0.420	0.437	0.449	0.526	0.516	0.450	0.459	4.431	1.729
ETTm1	96	**0.315**	**0.351**	0.365	0.387	0.353	0.370	0.379	0.419	0.386	0.398	0.505	0.475	0.672	0.571
	192	**0.355**	**0.375**	0.403	0.408	0.389	0.387	0.426	0.441	0.459	0.444	0.553	0.496	0.795	0.669
	336	**0.382**	**0.398**	0.436	0.431	0.421	0.408	0.445	0.459	0.495	0.464	0.621	0.537	1.212	0.871
	720	**0.441**	**0.432**	0.489	0.462	0.481	0.441	0.543	0.490	0.585	0.516	0.671	0.561	1.166	0.823
	Avg	**0.373**	**0.389**	0.423	0.422	0.411	0.402	0.448	0.452	0.481	0.456	0.588	0.517	0.961	0.734
ETTm2	96	**0.171**	**0.255**	0.197	0.296	0.183	0.266	0.203	0.287	0.192	0.274	0.255	0.339	0.365	0.453
	192	**0.232**	**0.294**	0.284	0.361	0.248	0.305	0.269	0.328	0.280	0.339	0.281	0.340	0.533	0.563
	336	**0.291**	**0.335**	0.381	0.429	0.309	0.343	0.325	0.366	0.334	0.361	0.339	0.372	1.363	0.887
	720	**0.382**	**0.390**	0.549	0.522	0.410	0.400	0.421	0.415	0.417	0.413	0.433	0.432	3.379	1.338
	Avg	**0.269**	**0.319**	0.353	0.402	0.287	0.329	0.305	0.349	0.306	0.347	0.327	0.371	1.410	0.810
Weather	96	0.179	0.232	0.198	0.261	0.195	0.236	0.217	0.296	**0.173**	**0.223**	0.266	0.336	0.300	0.384
	192	**0.221**	**0.264**	0.239	0.299	0.239	0.271	0.276	0.336	0.245	0.285	0.307	0.367	0.598	0.544
	336	**0.265**	**0.304**	0.288	0.336	0.289	0.306	0.339	0.380	0.321	0.338	0.359	0.395	0.578	0.523
	720	**0.349**	0.358	0.351	0.388	0.361	**0.351**	0.403	0.428	0.414	0.410	0.419	0.428	1.059	0.741
	Avg	**0.254**	**0.290**	0.268	0.321	0.271	0.291	0.309	0.360	0.288	0.314	0.338	0.382	0.634	0.548
Electricity	96	**0.165**	**0.257**	0.180	0.293	0.198	0.274	0.193	0.308	0.169	0.273	0.201	0.317	0.274	0.368
	192	**0.173**	**0.263**	0.189	0.302	0.198	0.278	0.201	0.315	0.182	0.286	0.222	0.334	0.296	0.386
	336	**0.182**	**0.273**	0.198	0.312	0.217	0.300	0.214	0.329	0.200	0.304	0.231	0.443	0.300	0.394
	720	**0.219**	**0.308**	0.217	0.330	0.278	0.356	0.246	0.355	0.222	0.321	0.254	0.361	0.373	0.439
	Avg	**0.185**	**0.275**	0.196	0.309	0.223	0.302	0.214	0.327	0.193	0.296	0.227	0.338	0.311	0.397
Traffic	96	**0.470**	**0.287**	0.577	0.350	0.647	0.384	0.587	0.366	0.612	0.338	0.613	0.388	0.719	0.391
	192	**0.479**	**0.292**	0.589	0.356	0.600	0.361	0.604	0.373	0.613	0.340	0.616	0.382	0.696	0.379
	336	**0.492**	**0.294**	0.594	0.358	0.610	0.367	0.621	0.383	0.618	0.328	0.622	0.337	0.777	0.420
	720	**0.501**	**0.309**	0.613	0.361	0.691	0.425	0.626	0.382	0.653	0.355	0.660	0.408	0.864	0.472
	Avg	**0.486**	**0.296**	0.593	0.356	0.637	0.384	0.610	0.376	0.624	0.340	0.628	0.379	0.764	0.416
Solar Energy	96	**0.186**	**0.256**	0.257	0.325	0.333	0.350	0.286	0.341	0.321	0.380	0.456	0.446	0.287	0.323
	192	**0.218**	**0.279**	0.278	0.354	0.371	0.372	0.291	0.337	0.346	0.369	0.588	0.561	0.297	0.341
	336	**0.227**	**0.288**	0.298	0.375	0.408	0.385	0.354	0.416	0.357	0.387	0.595	0.588	0.367	0.429
	720	**0.219**	**0.281**	0.299	0.379	0.406	0.377	0.380	0.437	0.375	0.424	0.733	0.633	0.374	0.431
	Avg	**0.213**	**0.276**	0.283	0.358	0.380	0.371	0.328	0.383	0.350	0.390	0.586	0.557	0.331	0.381

**Table 4:** Ablation study of the UP Loss components on all datasets. We compare the baseline (MSE) against variants using only temporal weight ($w_{\mathrm{time}}$), only complexity weight ($w_{\mathrm{complexity}}$), and the full UP Loss. Best results are in bold.

Dataset	Horizon	Metric	w/o All	w/$w_{\mathrm{time}}$	w/$w_{\mathrm{complexity}}$	UP-TimeMixer
ETTh1	96	MSE	0.375	0.374	0.373	**0.371**
		MAE	0.400	0.398	0.397	**0.395**
	720	MSE	0.498	0.485	0.491	**0.479**
		MAE	0.482	0.472	0.478	**0.465**
ETTh2	96	MSE	0.289	0.287	0.286	**0.285**
		MAE	0.341	0.340	0.339	**0.338**
	720	MSE	0.412	0.407	0.409	**0.405**
		MAE	0.434	0.430	0.432	**0.428**
ETTm1	96	MSE	0.320	0.317	0.318	**0.315**
		MAE	0.357	0.353	0.354	**0.351**
	720	MSE	0.454	0.448	0.445	**0.441**
		MAE	0.441	0.437	0.435	**0.432**
ETTm2	96	MSE	0.175	0.173	0.172	**0.171**
		MAE	0.258	0.257	0.256	**0.255**
	720	MSE	0.391	0.385	0.387	**0.382**
		MAE	0.396	0.392	0.394	**0.390**
Weather	96	MSE	**0.163**	0.170	0.172	0.179
		MAE	**0.209**	0.220	0.224	0.232
	720	MSE	**0.339**	0.346	0.343	0.349
		MAE	**0.341**	0.352	0.348	0.358
Electricity	96	MSE	**0.153**	0.159	0.161	0.165
		MAE	**0.247**	0.252	0.254	0.257
	720	MSE	0.225	0.222	0.221	**0.219**
		MAE	0.310	0.309	0.310	**0.308**
Traffic	96	MSE	**0.462**	0.466	0.468	0.470
		MAE	**0.285**	0.286	0.287	0.287
	720	MSE	0.506	0.504	0.503	**0.501**
		MAE	0.313	0.311	0.310	**0.309**
Solar-Energy	96	MSE	0.189	0.188	0.187	**0.186**
		MAE	0.259	0.258	0.257	**0.256**
	720	MSE	0.223	0.221	0.220	**0.219**
		MAE	0.285	0.283	0.282	**0.281**

**Table 5:** Parameter sensitivity analysis of λ on the ETTh1 dataset (Avg. MSE/MAE over 4 horizons).

Metric	Baseline (TimeMixer)	λ=0.1	λ=0.3	λ=0.5	λ=0.7	λ=0.9
MSE	0.447	0.443	0.439	0.436	0.441	0.445
MAE	0.440	0.438	0.434	0.431	0.435	0.439

## Data Availability

The ETT, Electricity, Traffic, Weather, and Solar-Energy datasets can be found at the following repositories: https://github.com/zhouhaoyi/ETDataset (access on 25 November 2025) and https://github.com/thuml/Autoformer (access on 25 November 2025).
